# A highly sensitive and selective hydroquinone sensor based on a newly designed N-rGO/SrZrO_3_ composite†

**DOI:** 10.1039/c9na00573k

**Published:** 2019-12-09

**Authors:** Khursheed Ahmad, Praveen Kumar, Shaikh M. Mobin

**Affiliations:** Discipline of Chemistry, Indian Institute of Technology Indore Simrol, Khandwa Road Indore 453552 India xray@iiti.ac.in +91 731 2438 752; Discipline of Biosciences and Bio-Medical Engineering, Indian Institute of Technology Indore Simrol, Khandwa Road Indore 453552 India; Discipline of Metallurgy Engineering and Materials Science, Indian Institute of Technology Indore Simrol, Khandwa Road Indore 453552 India

## Abstract

Herein, we have reported a novel composite of nitrogen doped reduced graphene oxide (N-rGO) and strontium zirconate (SrZrO_3_). This new composite (N-rGO/SrZrO_3_) was synthesized using the reflux method. The physicochemical properties of N-rGO/SrZrO_3_ were determined using different advanced techniques such XRD, FE-SEM, EDX, FTIR and BET. Furthermore, a glassy carbon electrode was modified with N-rGO/SrZrO_3_ (GCE-2). This modified electrode was employed for the sensing of HQ. The electrochemically active surface area (ECSA) of this modified electrode (GCE-2) was calculated by employing the Randles–Sevcik equation. Furthermore, GCE-2 exhibited a good detection limit (0.61 μM) including high selectivity towards HQ.

## Abstract 2

Herein, we have reported a novel composite of nitrogen doped reduced graphene oxide (N-rGO) and strontium zirconate (SrZrO_3_).

## Graphical abstract

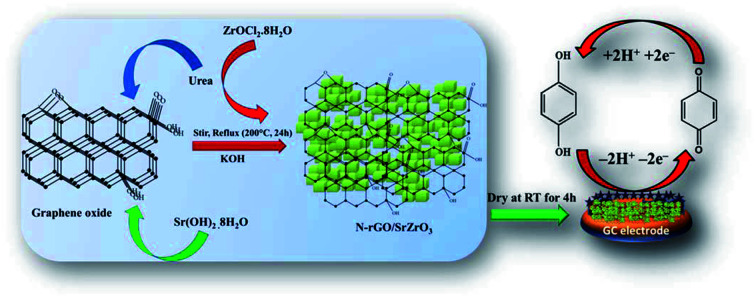

## 1. Introduction

Hydroquinone (HQ) which is a di-substituted phenol (1,4-dihydroxybenzene) compound has been extensively employed in various fields (dyes, cosmetics, photo-stabilizers, pharmaceuticals, oil-refinery, plasticizers, pesticides, textile *etc.*).^1–3^ HQ exists in the environment as a toxic pollutant and its degradation is difficult under ecological circumstances.^4,5^ Moreover, HQ has negative impacts on the environment and human health.^5^ Even trace amounts of HQ may also cause hypoesthesia, puking, debility, fatigue, kidney damage and headache.^6–9^ HQ is widely used in cosmetics for bleaching purposes but long-term/excessive use may be responsible for skin damage or severe allergy.^6^ Thus, a sensitive and reliable method is required to detect trace levels of HQ. In last few decades, various approaches and methods (chemiluminescence, flow injection, pulse radiolysis, high performance liquid chromatography, synchronous fluorescence, electrochemical and solid-phase extraction) have been widely used for the determination of HQ.^10–16^ Among these methods, the electrochemical approach has emerged as a most promising detection technique due to its simplicity, cost effectiveness, ease of handling, selectivity and high sensitivity.^17,18^ Various novel electrode materials have been employed for the electrochemical detection of HQ. Previously, Qi *et al.* prepared a novel S,N doped 3D graphene for the sensing of phenol derivatives.^18^ Gan *et al.* employed a hybrid composite of graphene oxide and MnO_2_ whereas Peng *et al.*^19^ used a MoS_2_/reduced graphene oxide composite for the sensing of HQ.^20^ Although electrochemical techniques are the most promising approach, their performance largely depends on the electrode materials (electro-catalysts). The charge transfer, presence of surface electrochemically active sites and surface morphology also influence the performance of the electrochemical sensing devices.^18^ In some cases, transition metal oxides may suffer from poor conductivity and this phenomenon restricts their electrochemical applications. To address such issues, reduced graphene has been employed as a promising conductive support for poorly semiconducting metal oxides.^19^ Moreover, some research groups created active sites inside reduced graphene oxide by doping atoms into the graphene matrix.^18^ Therefore, it is of great interest to design and develop new electrode materials with a unique surface morphology for electrochemical sensors.

Perovskite materials with the general formula ABO_3_ possess excellent physicochemical properties such as ferroelectric, magnetic and two-dimensional electrical conductivity.^21,22^ In last few years, ABO_3_ perovskites have been explored in various applications such as photovoltaics, catalysis, batteries, opto-electronics, super-capacitors, fuel cells, sensors *etc.*^23–25^ Recently, among zirconium based perovskites such as BaZrO_3_, CaZrO_3_, and SrZrO_3_ perovskites, ferroelectric strontium zirconate (SrZrO_3_) has attracted much attention owing to its outstanding thermo-mechanical and electrical properties.^26^ It has a highly stable nature at high temperature and acts as a sensor. Previously, the SrZrO_3_ perovskite had been employed as an electrode material for water splitting applications and also been proposed as future materials for non-volatile memory applications.^27^ Thus, SrZrO_3_ has potential for electrochemical applications. For the last few years, our research group has been working on hybrid materials for dye sensitized solar cells, electrochemical sensors, perovskite solar cells and water splitting applications.^28–33^

**Scheme 1:**
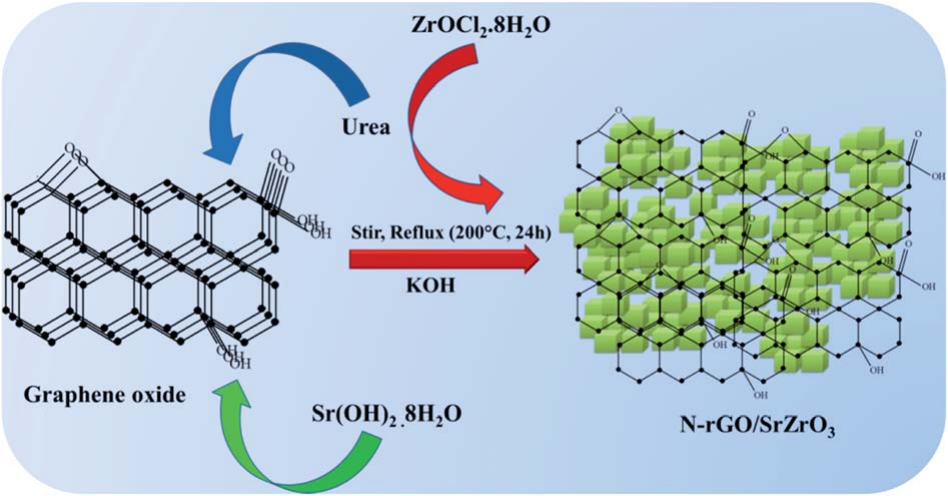
Schematic representation of the synthesis of the N-rGO/SrZrO_3_ composite.

Herein, for the first time, a novel composite of SrZrO_3_ cubes embedded on nitrogen doped reduced graphene oxide (N-rGO/SrZrO_3_) has been synthesized for the electrochemical sensing of HQ (Scheme 1).

## 2. Materials and methods

### 2.1 Chemicals and reagents

Phosphate buffered saline (PBS) solutions, ZrOCl_2_·8H_2_O, graphite, urea, Sr(OH)_2_·8H_2_O, potassium hydroxide, hydroquinone and other used chemicals/reagents/solvents were purchased from Alfa Aesar, Loba, Fisher Scientific, TCI, SRL and Merck, India.

### 2.2 Synthesis of the N-rGO/SrZrO_3_ composite

Graphene oxide (GO) was synthesized according to our previous report.^33^ 50 mg of GO was dispersed in deionized (D.I.) water and sonicated for 2 h at RT. 1.0 gm of urea was added to the GO and stirred for 30 min. 0.48 gm of Sr(OH)_2_·8H_2_O was added to the GO dispersion, followed by the addition of 0.538 gm of ZrOCl_2_·8H_2_O with continuous stirring at RT. Furthermore, 10 gm of potassium hydroxide (KOH) dissolved in DI water was added slowly to the precursor solution and the stirring continued for 24 h at 200 °C under reflux (Scheme 1). Finally, the obtained precipitate was washed with diluted acetic acid and water to remove the residues and dried overnight at 60 °C.

### 2.3 Fabrication of the N-rGO/SrZrO_3_ composite modified glassy carbon electrode

The bare GCE is denoted as GCE-1. A glassy carbon electrode (GCE) was polished using alumina slurry and sonicated for 30 minutes, followed by drop casting of 8 μL of the N-rGO/SrZrO_3_ composite (GCE-2) with 0.1% Nafion onto the active surface area of the GCE (3 mm) which was further kept to dry at RT for 4 h. For control experiments, the active area of the GCE was also drop casted with SrZrO_3_ (GCE-3) and N-rGO (GCE-4) followed by Nafion deposition.

## 3. Results and discussion

### 3.1 General characterization of N-rGO/SrZrO_3_

**Fig. 1:**
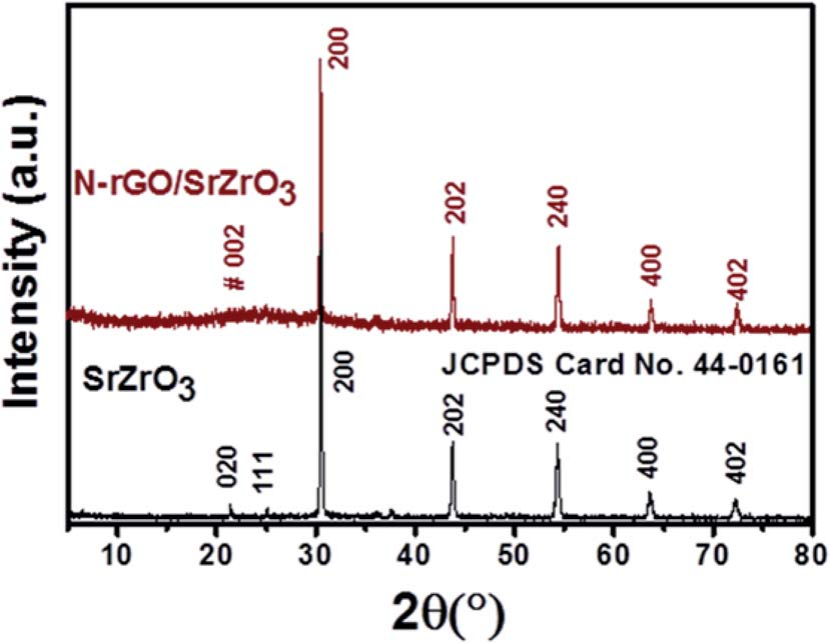
PXRD patterns of SrZrO_3_ (black) and N-rGO/SrZrO_3_ (brown).

Powder X-ray diffraction (XRD) measurements were performed to understand and confirm the formation of the prepared samples (SrZrO_3_, N-rGO and N-rGO/SrZrO_3_ composite). The XRD patterns of SrZrO_3_ (black) and the N-rGO/SrZrO_3_ composite (brown) were recorded in the 2*θ* range of 10–80° and are presented in Fig. 1. In the case of SrZrO_3_, the diffraction peaks were observed at 21.4°, 25.01°, 30.6°, 43.75°, 54.15°, 63.53° and 72.27° which corresponded to the (020), (111), (200), (202), (240), (400) and (402) planes respectively. The XRD examinations confirmed the formation of SrZrO_3_ and all the diffraction peaks were found to be well-matched with previous JCPDS card no 44-0161. Moreover, the high intensity of the diffraction peaks suggested the crystalline nature of the prepared SrZrO_3_.

In the case of the hybrid composite composed of N-rGO and SrZrO_3_, similar diffraction planes were observed. However, a broader diffraction peak appearing at ∼23.91° was also observed which was attributed to the (002) plane of N-rGO. Therefore, the XRD investigations suggested the successful formation of the N-rGO/SrZrO_3_ composite. However, the two (020) and (111) diffraction planes were absent in the XRD pattern of N-rGO/SrZrO_3_ which may be due to the broad diffraction peak and amorphous nature of rGO. The crystallite size of the prepared SrZrO_3_ and N-rGO/SrZrO_3_ composite was determined by employing the Debye–Scherrer equation given below:1
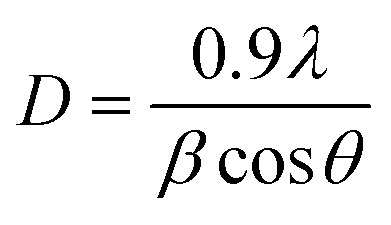


In eqn (1), *β* = broadening of the diffraction line and *λ* = 2.2897 Å. The crystallite size of SrZrO_3_ and the N-rGO/SrZrO_3_ composite was found to be 51.1 nm and 43.6 nm respectively. The other crystal parameters are shown in Table S1.† Furthermore, the micro strain (*ε*) was also calculated using the following equation according to previous reports:^34–36^2
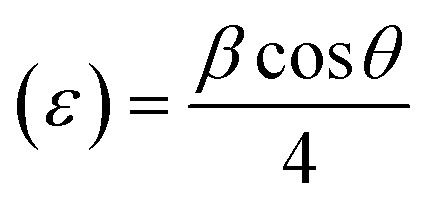


The *ε* was deduced to be 0.040 and 0.047 for SrZrO_3_ and N-rGO/SrZrO_3_ respectively. The dislocation density (*δ*) was also calculated using the following formula:

3 Dislocation density (*δ*) = 1/*D*^2^

The dislocation density was calculated to be 0.000382 and 0.000526 for SrZrO_3_ and N-rGO/SrZrO_3_ respectively. The XRD pattern of the separately prepared N-rGO was also recorded and is presented in Fig. S1.† The recorded XRD pattern exhibited a broad diffraction peak between 20° to 30° which confirmed the formation of N-rGO.

**Fig. 2:**
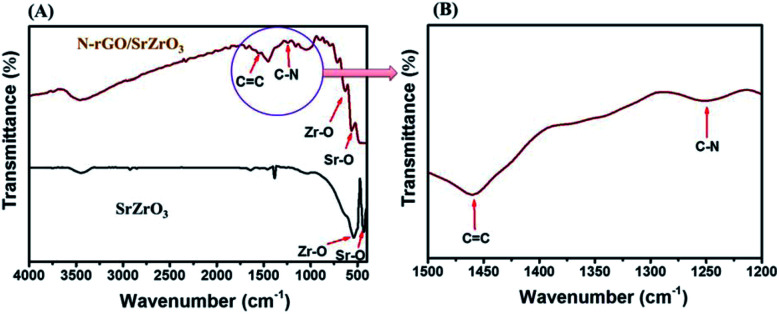
(A) FTIR spectra of SrZrO_3_ (black) and N-rGO/SrZrO_3_ (brown). (B) Enlarged view.

Fourier-transform infrared (FTIR) analysis was also conducted to understand the presence of chemical bonds in SrZrO_3_ and the N-rGO/SrZrO_3_ composite. The obtained FTIR spectra of SrZrO_3_ (black) and N-rGO/SrZrO_3_ (brown) are displayed in Fig. 2.

**Fig. 3:**
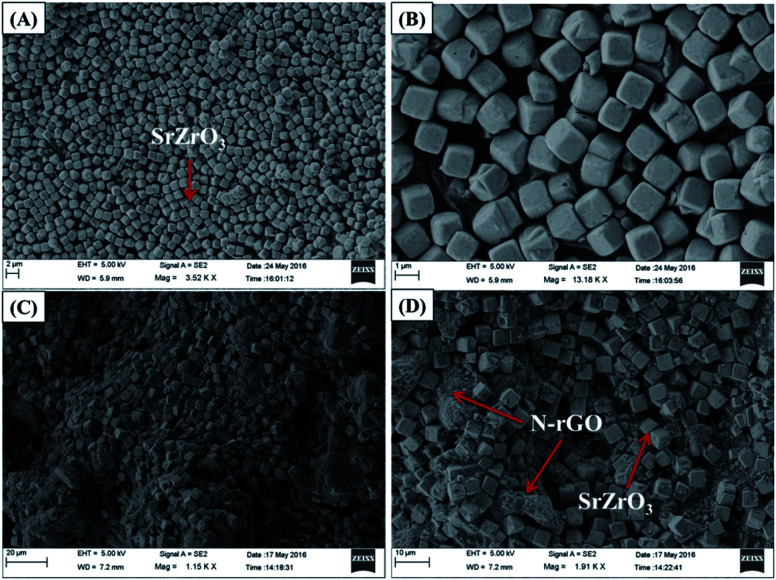
(A and B) FE-SEM images of SrZrO_3_ and (C and D) N-rGO/SrZrO_3_.

In the case of SrZrO_3_, two adsorption bands were observed at 440 cm^−1^ and 533.7 cm^−1^. These two bands were assigned to the presence of Sr–O and Zr–O stretching vibration modes in SrZrO_3_ (black) respectively (Fig. 2A). The FTIR spectrum of the N-rGO/SrZrO_3_ composite showed adsorption bands at 568.1 cm^−1^ and 622.3 cm^−1^ which were assigned to the Sr–O and Zr–O stretching vibration modes whereas the other adsorption bands at 1244.1 cm^−1^ and 1559.6 cm^−1^ suggested the presence of C–N and C=C bonds respectively. The FTIR analysis confirmed the formation of the N-rGO/SrZrO_3_ composite (brown) as shown in Fig. 2A (brown). The FTIR spectra of N-rGO are presented in Fig. S2† which showed C=C and C–N bonds. The C–N bond confirmed the doping of N atoms into the rGO framework. The particle surface morphology of the prepared SrZrO_3_ and N-rGO/SrZrO_3_ composite was determined by FE-SEM analysis and the recorded images are shown in Fig. 3.

**Fig. 4:**
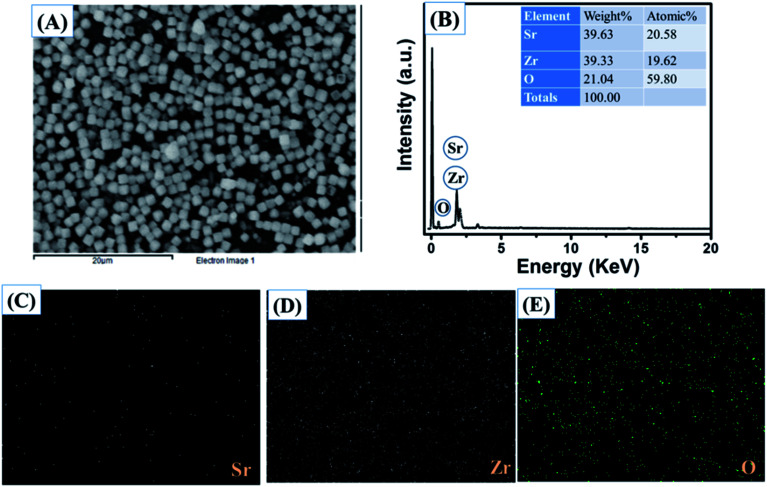
(A) Electron image, (B) EDX spectrum and (C–E) EDX mapping images of SrZrO_3_.

**Fig. 5:**
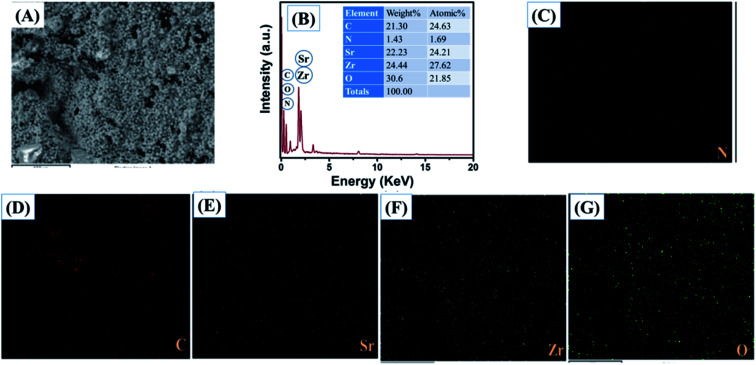
(A) Electron image, (B) EDX spectrum and (C–G) EDX mapping images of N-rGO/SrZrO_3_.

The obtained results clearly showed that the prepared SrZrO_3_ has a cube like surface morphology (Fig. 3A and B). Furthermore, the recorded FE-SEM images of the N-rGO/SrZrO_3_ composite also showed that the SrZrO_3_ cubes are embedded in the 3D-network of N-rGO sheets resulting in the N-rGO/SrZrO_3_ composite (Fig. 3C and D). The FE-SEM analysis of the prepared N-rGO showed a sheet like surface morphology and the recorded FE-SEM images are shown in Fig. S3.† The elemental composition analysis of a hybrid composite is a crucial factor which also suggests the formation of the composites with the presence of elements. The recorded EDX spectra and EDX mapping images of SrZrO_3_ are presented in Fig. 4. The obtained EDX spectrum showed the presence of Sr, Zr and O elements which confirmed the formation of SrZrO_3_ (Fig. 4B). The EDX mapping images of the Sr, Zr and O elements are displayed in Fig. 4C and D. The EDX spectrum and mapping images of the N-rGO/SrZrO_3_ composite are presented in Fig. 5 which showed the presence of N, C, Sr, Zr, and O (Fig. 5B) and suggested the formation of the N-rGO/SrZrO_3_ composite (Fig. 5C–G).

### 3.2 Cyclic voltammetry investigations

First, the electrochemical performance of GCE-2 was compared with differently modified electrodes (GCE-3; GCE-4) and bare GCE (GCE-1) in the presence of 250 μM hydroquinone (HQ) in 0.1 M PBS (pH 7.0) at an applied scan rate of 100 mV s^−1^ by employing the CV method.

**Fig. 6:**
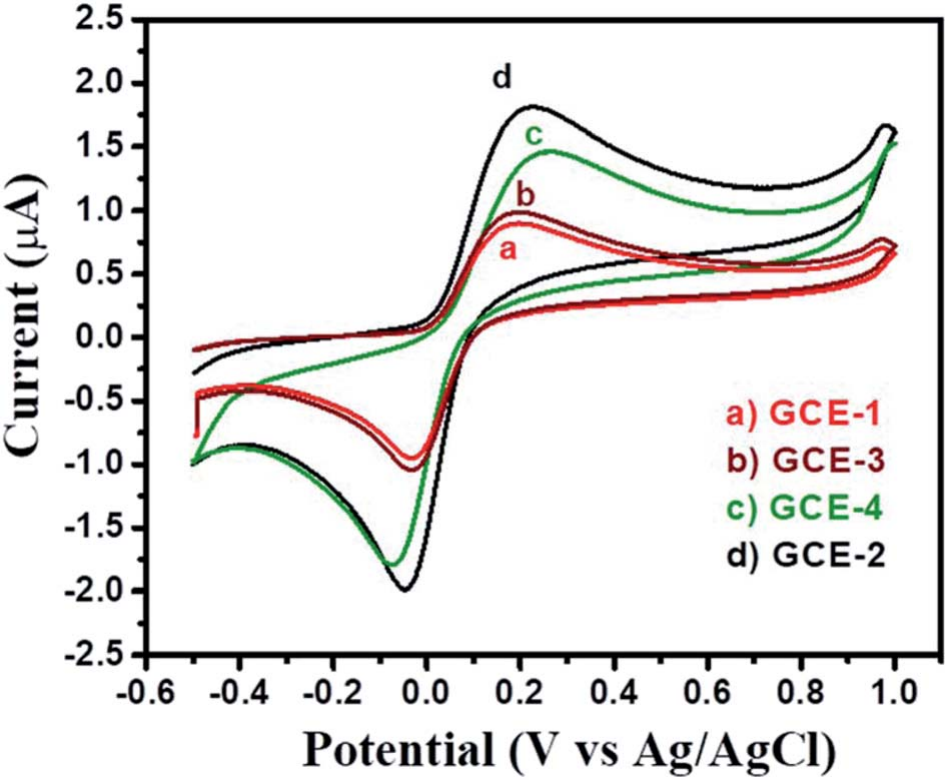
CV curves of bare GCE-1 (red), GCE-2 (black), GCE-3 (brown) and GCE-4 (green) in the presence of 250 μM HQ in 0.1 M PBS at a scan rate of 100 mV s^−1^.

The CV curves are presented in Fig. 6 which showed that the lowest current was obtained for GCE-1 whereas GCE-2 showed highest current compared to GCE-3 and GCE-4. Thus, it can be clearly understood that GCE-2 has good electrocatalytic characteristics towards HQ which may be due to the synergistic effects between N-rGO and SrZrO_3_ and the high surface area of N-rGO/SrZrO_3_ (54.2 m^2^ g^−1^) compared to pristine SrZrO_3_ (32.9 m^2^ g^−1^). The N_2_ adsorption–desorption isotherms of SrZrO_3_ and N-rGO/SrZrO_3_ are shown in Fig. S4.†

The electrochemically active surface area (ECSA) of GCE-1 and modified GC electrodes (GCE-2, GCE-3 and GCE-4) was calculated by recording CV curves (Fig. S5†) in the [Fe(CN)_6_]^3−/4−^ redox couple by employing the Randles–Sevcik equation:^37^

4 *I*_p_ = 2.69 × 10^5^*AD*^1/2^*n*^3/2^*y*^1/2^C

where *I*_p_ is the peak current, *A* = ECSA (to be calculated), *y* is the scan rate (V s^−1^), *n* = no. of electrons (*n* = 1), *C* is the concentration (mol L^−1^) of [Fe(CN)_6_]^3−/4−^ (redox couple) and *D* is the diffusion coefficient (6.7 × 10^−6^ cm^2^ s^−1^). The ECSA of GCE-1, GCE-2, GCE-3 and GCE-4 was found to be 0.07 cm^2^, 0.22 cm^2^, 0.092 cm^2^ and 0.179 cm^2^ respectively. To check the electrocatalytic activity and charge transfer characteristics of GCE-1, GCE-2, GCE-3 and GCE-4, they were investigated by using electrochemical impedance spectroscopy (EIS). The recorded EIS curves (Nyquist) of GCE-1, GCE-2, GCE-3 and GCE-4 in 5 mM redox probe [Fe(CN)_6_]^3/4^ are presented in Fig. S6.† The equivalent circuit is presented in the inset of Fig. S6.† From Fig. S6† it can be seen that GCE-1 has a larger semi-circle whereas a small semi-circle was observed for GCE-2. This exhibited the lower charge resistance for GCE-2 compared to the other modified electrodes (GCE-1, GCE-3 and GCE-4). It is well known that a higher charge resistance restricts/limits the electron transfer reactions between the electrode and analytes. Thus, GCE-1 showed a poor current response whereas GCE-2 with the smallest charge resistance showed the highest current response. The charge resistance values for GCE-1, GCE-2, GCE-3 and GCE-4 are summarized in Table S2.† The pH of the analyte solution plays an important role and affects the performance of the modified electrodes. Thus we have checked the effect of pH on the performance of GCE-2 towards HQ by recording CV curves in the presence of 250 μM HQ at different pH values (2, 4, 7, 9 and 10) with a constant scan rate of 100 mV s^−1^.

**Fig. 7:**
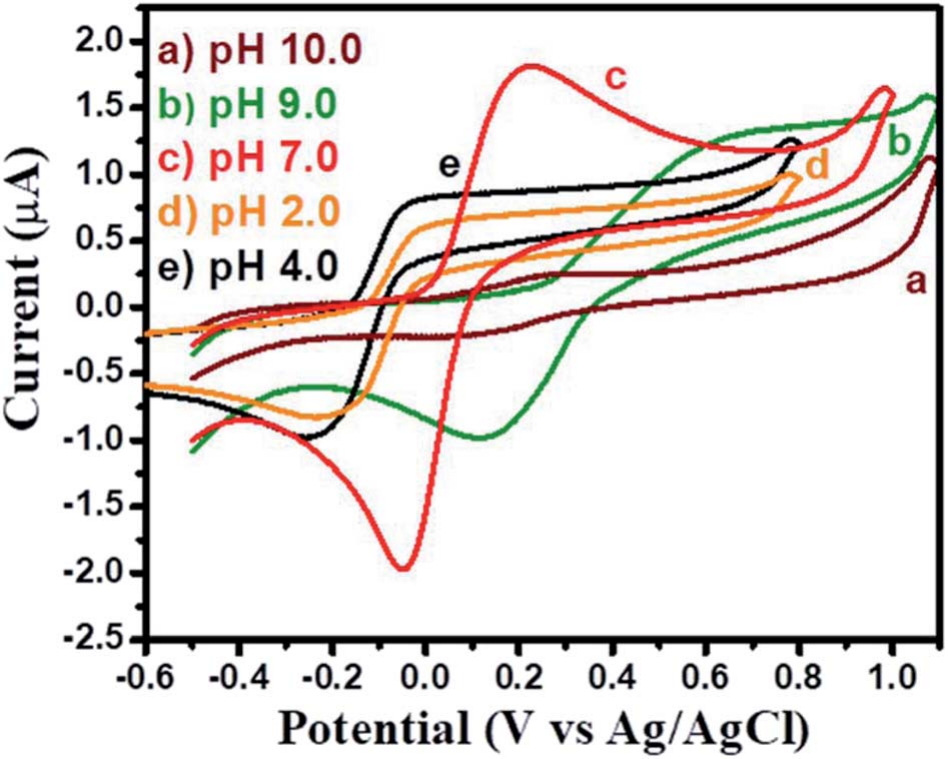
CV curves of GCE-2 in the presence of 250 μM HQ in 0.1 M PBS at a scan rate of 100 mV s^−1^ at different pH values.

The obtained CV results for GCE-2 exhibited the highest current response at pH = 7.0 and this pH was used for further electrochemical investigations (Fig. 7).

**Fig. 8:**
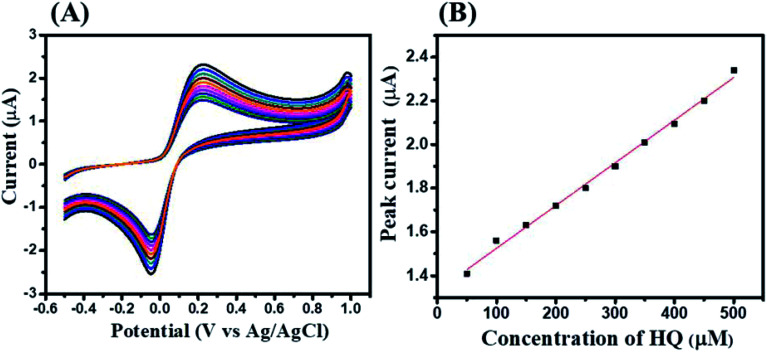
CV curves of GCE-2 (A) in the presence of different concentrations of HQ (50–500 μM) in 0.1 M PBS (pH 7.0) with a scan rate of 100 mV s^−1^ and the calibration plot of peak current *versus* concentration (B).

To check the effect of concentrations on the electrocatalytic activity of GCE-2, CV curves were recorded by varying the concentration (50–500 μM) of HQ at optimized pH at a scan rate of 100 mV s^−1^ (Fig. 8A).

The obtained results showed that the electrocatalytic current increases with increasing concentration of HQ. The calibration curve of the peak current against the concentration of HQ is plotted in Fig. 8B which showed a linearly increase in the current response.

**Fig. 9:**
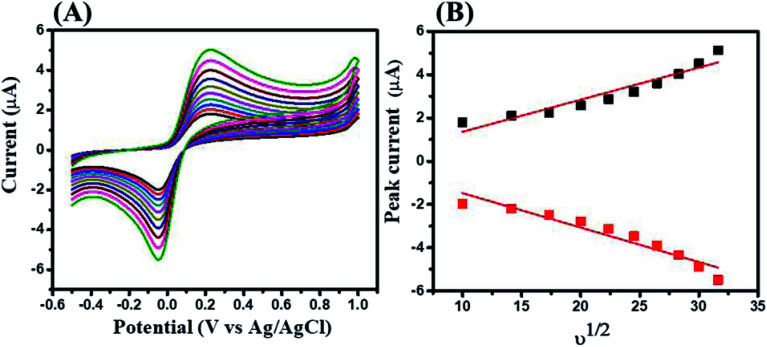
CV curves of GCE-2 (A) at different scan rates (100–1000 mV s^−1^) in the presence of 250 μM HQ in 0.1 M PBS (pH 7.0) and the calibration plot of peak current *versus* square root of the scan rate (B).

Furthermore, the effect of scan rate on the electrocatalytic activity of GCE-2 was also investigated by tuning the scan rate (100–1000 mV s^−1^) in the presence of 250 μM HQ in 0.1 M PBS at pH 7.0 (Fig. 9A). The obtained results suggested that the electrocatalytic current response increased linearly upon increasing the scan rate as plotted in the calibration curve of the peak current *versus* square root of the scan rate (Fig. 9B). This linearly increased current suggested a diffusion controlled process.

### 3.3 Square wave voltammetry investigations

**Fig. 10:**
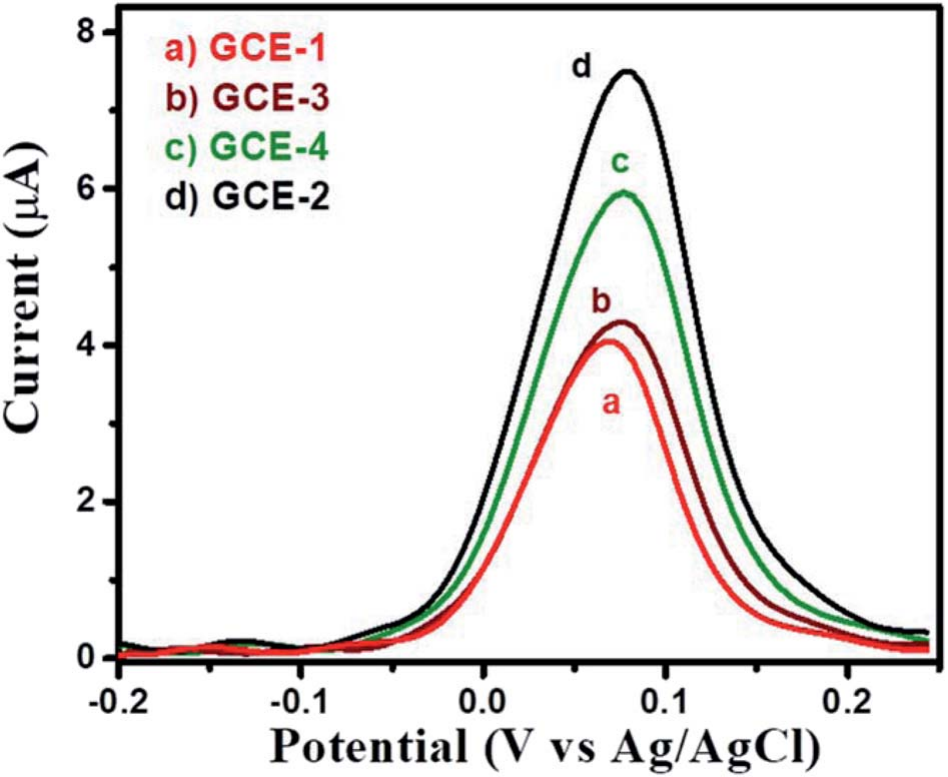
SWV curves of GCE-1 (red), GCE-2 (black), GCE-3 (brown) and GCE-4 (green) in the presence of 250 μM HQ in 0.1 M PBS (pH = 7.0).

The square wave voltammetry (SWV) approach has been known for its higher sensitivity compared to cyclic voltammetry. Thus, we have employed the SWV electrochemical approach for the electrochemical sensing of HQ. SWV curves were recorded for GCE-1, GCE-2, GCE-3 and GCE-4 in the presence of 250 μM HQ in 0.1 M PBS (pH = 7.0). The observations revealed that a higher current response was obtained for GCE-2 (Fig. 10) compared to the other electrodes (GCE-1, GCE-3 and GCE-4).

**Fig. 11:**
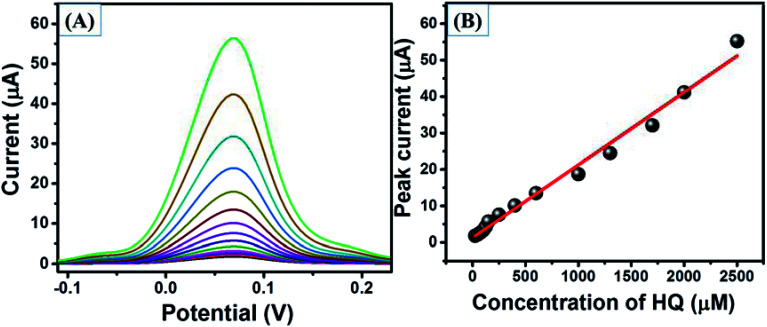
SWV curves (A) of GCE-2 in the presence of different concentrations of HQ and the calibration plot (B) of peak current *versus* concentrations of HQ.

This enhanced current may be due to the synergy effect, high specific surface area (Fig. S4†) and electrochemical surface area (Fig. S5†) of N-rGO/SrZrO_3_ compared to pristine SrZrO_3_. Furthermore, the SWV curves of GCE-2 were also recorded in the presence of different concentrations (25 μM, 50 μM, 75 μM, 100 μM, 125 μM, 150 μM, 250 μM, 400 μM, 600 μM, 1000 μM, 1300 μM, 1700 μM, 2000 μM and 2500 μM) of HQ (Fig. 11A). The obtained results showed that the current response increases with increasing concentration of HQ. The calibration plot was plotted between the peak current and concentrations of HQ which revealed that the current increases in a linear way (Fig. 11B).

**Scheme 2:**
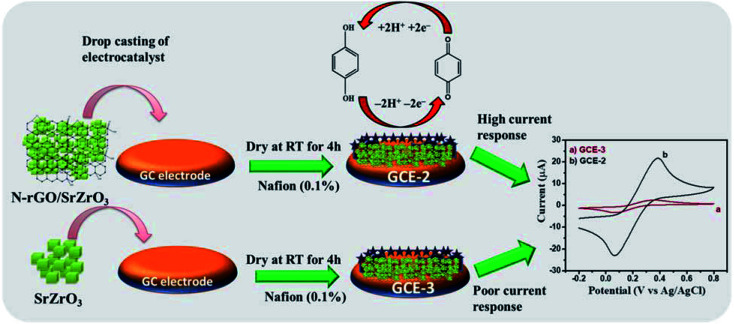
Schematic illustration of the electrochemical sensing of HQ.

The probable mechanism of the sensing of HQ has been adopted from previous literature.^7^ The electrochemical detection of HQ involves the reversible oxidation–reduction reaction as suggested by CV investigations. The two bonds (oxygen–hydrogen) of the phenolic hydroxyl groups broke and in the meantime, HQ lost two electrons and two protons and converted to the quinoid structure (Scheme 2).

Therefore it is clear that the first step involves the conversion of HQ to quinone by releasing 2 protons and subsequently, reverting to HQ by accepting the released protons as depicted in Scheme 2.

### 3.4 Selectivity

**Fig. 12:**
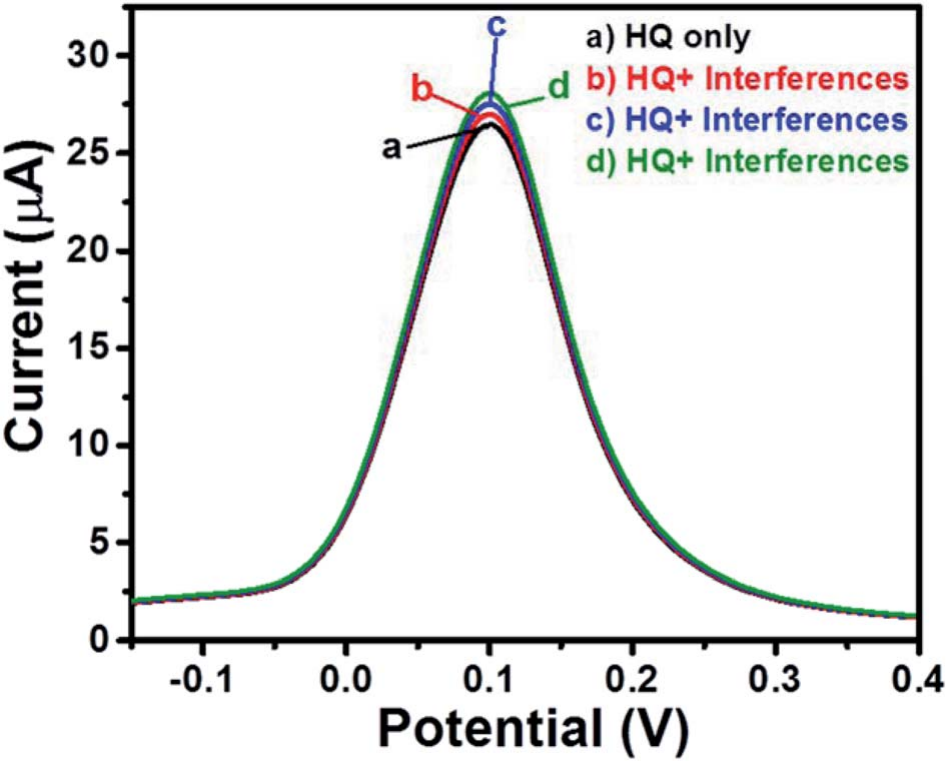
SWV curves of GCE-2 in the presence of different interfering species.

Selectivity is one of the most important characteristics of any ideal sensor. There are many interfering species which may cause an interfering atmosphere which results in inaccurate determination of the analyte. Thus, we have checked the selectivity of GCE-2 towards HQ sensing by using the SWV method. Initially, we have recorded the SWV curve of GCE-2 in the presence of 1400 μM HQ (Fig. 12a). Furthermore, 2500 μL of glucose + fructose was injected and the SWV curve was also recorded (Fig. 12b). Subsequently, 1500 μL of hydrazine + Mg^2+^ (Fig. 12c) and 1500 μL of dopamine + uric acid + ascorbic acid (Fig. 12d) were also injected and the recorded SWV curves revealed insignificant variation in the current response which indicated the higher selectivity of GCE-2 towards HQ.

### 3.5 Repeatability and stability

The repeatability of GCE-2 for the sensing of HQ was also evaluated by recording 5 consecutive SWV curves in the presence of 1600 μM HQ in 0.1 M PBS at pH 7.0 using the SWV method (Fig. S7†). The relative standard deviation (RSD) of the peak current was found to be 2.67%. Subsequently, to check the stability, GCE-2 was kept for 18 days in a vacuum desiccator and the current response was checked using SWV and an insignificant change was observed which confirms its stability for long-term use.

### 3.6 Comparison

The detection limit was calculated to be 0.61 μM using the equation 3.3 × *σ*/slope (where *σ* is the standard deviation). Dang *et al.*^7^ employed a CePO_4_/CPE sensor to detect HQ which showed a detection limit of 0.70 μM. Zhang *et al.*^38^ employed a polarized glassy carbon electrode which showed a detection limit of 3.7 μM. Carboxylic functional multi-walled carbon nanotubes have been used to construct a HQ sensor by Feng *et al.*^39^ and the developed sensor has shown a detection limit of 2.3 μM. We have compared the detection limit obtained by GCE-2 with previously reported sensors and the results are summarized in Table 1. From Table 1, it is clear that GCE-2 has advantages over previously reported sensors in terms of the detection limit and wide linear range.

**Table 1:** Comparison of GCE-2 with previously reported HQ sensors

No.	Electrode	Linear range (μA μM^−1^)	Limit of detection (LOD) μM	References
1.	CePO_4_/CPE	0.4–50	0.70	7
2.	Polarized glassy carbon electrode	10–300	3.57	38
3.	Carboxylic functional multi-walled carbon nanotubes	10–120.0	2.3	39
4.	Carbon nanocages–RGO/GCE	1–400	0.87	40
5.	Poly-amidosulfonic acid/MWNTs/GCE	8–391	2.60	41
6.	MWCNT–poly-malachite green/GCE	10–480	1.60	42
7.	Carbon nano-fragment–AuNPs	9–500	0.86	43
8.	AuNPs/Fe_3_O_4_/APTESGO/GCE	3–137	1.1	44
**9.**	GCE-2	**25–2500**	**0.61**	***This work***

## 4. Conclusion

Finally, it can be concluded that a SrZrO_3_ cube embedded nitrogen doped reduced graphene oxide (N-rGO–SrZrO_3_) composite has been prepared by using the reflux method. Furthermore, a highly selective electrochemical sensor (hydroquinone sensor) was fabricated by employing N-rGO–SrZrO_3_ as the electrode modifier. Different electrochemical approaches (cyclic voltammetry and square wave voltammetry) have been employed for the determination of hydroquinone. The fabricated sensor has shown excellent performance towards the detection of hydroquinone using square wave voltammetry compared to cyclic voltammetry. Moreover, the fabricated sensor exhibited a good detection limit, repeatability, reproducibility and high selectivity towards the sensing of hydroquinone.

## Conflicts of interest

There are no conflicts to declare.

## Supplementary Material

NA-002-C9NA00573K-s001
