# Supplementary material for: A highly sensitive and selective hydroquinone sensor based on a newly designed N-rGO/SrZrO3 composite
Source: Nanoscale Adv. 2019 Dec 9;2(1):502–11. doi: 10.1039/c9na00573k (PMC9417952; doi:10.1039/c9na00573k)
Supplement: NA-002-C9NA00573K-s001 [file NA-002-C9NA00573K-s001.pdf]

Electronic Supplementary Material (ESI) for Nanoscale Advances.
This journal is © The Royal Society of Chemistry 2019

Supplementary Information

A highly sensitive and selective hydroquinone sensor based on a newly designed N-rGO/$SrZrO_3$ composite

Khursheed Ahmad,[a] Praveen Kumar [a] and Shaikh M. Mobin*[a,b,c]

[a]Discipline of Chemistry, [b]Discipline of Biosciences and Bio-Medical Engineering and [c]Discipline of Metallurgy Engineering and Materials Science, Indian Institute of Technology Indore, Simrol, Khandwa Road, Indore 453552, India

*Corresponding author: email: xray@iiti.ac.in

Tel: +91 731 2438 75 2

Instrumental

The powder x-ray diffraction patterns (XRD) were performed on a Rigaku, Japan, RINT 2500 V x-ray diffractometer with Cu Kα irradiation (λ = 1.5406 Å). The particle surface morphology and elemental constituents of the synthesized samples were characterized by using Supra 55 Zeiss Field Emission Scanning Electron microscope (FE-SEM) attached with Energy Dispersive X-ray (EDX) spectroscopy (Oxford Instrument' X-max, Aztec). The electrochemical measurements were performed on Metrohm Autolab PGSTAT 204N consists of three electrode system, using glassy carbon electrode as a working electrode, platinum wire as a counter electrode with Ag/AgCl as a reference electrode using NOVA software (version 1.10).

Experimental section

Synthesis of $SrZrO_3$ cubes

0.48gm of $Sr(OH)_2.8H_2O$ was dissolved in DI water and 0.538gm of $ZrOCl_2.8H_2O$ was added with continuous stirring at RT. Further, 10gm of potassium hydroxide (KOH) dissolved in D.I. water was added slowly to the above mixture and stirring continued for 24h at 200°C under reflux. Finally, the obtained precipitate was washed with diluted acetic acid and water to remove the residues and dried over night at 60°C.

Synthesis of N-rGO

50mg of GO was dispersed in deionized water (D.I.) and sonicated for 2h at RT. 1gm of urea was added into the GO stirred for 12h at 200°C under reflux. The obtained black precipitate was washed with ethanol, D.I. water and dried under vacuum.

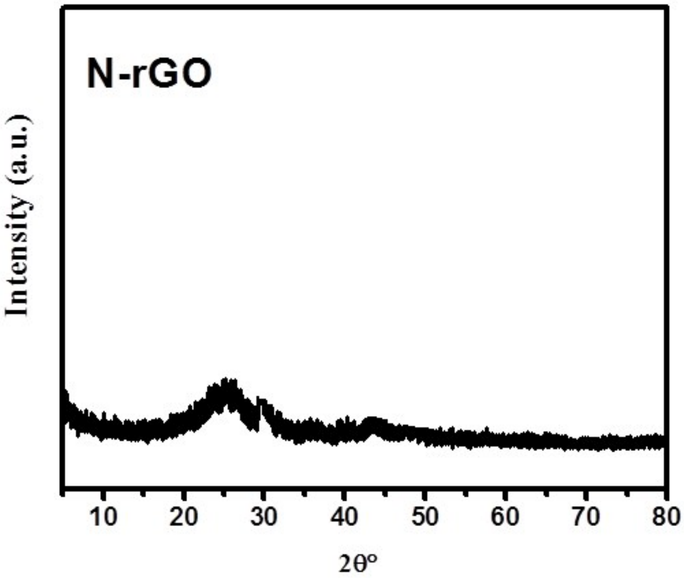

Fig. S1 XRD pattern of the N-rGO.

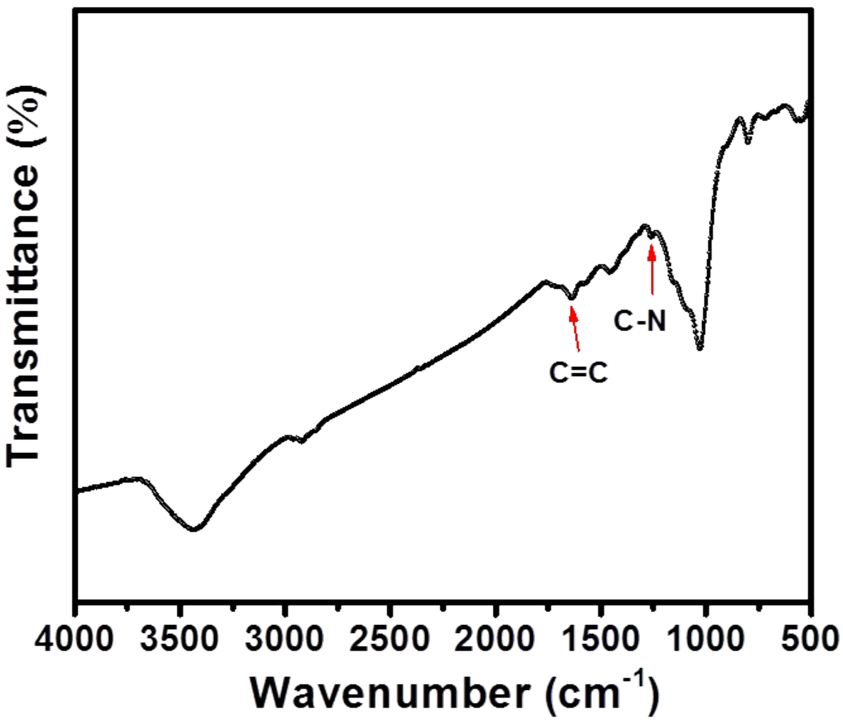

Fig. S2 FTIR spectra of N-rGO.

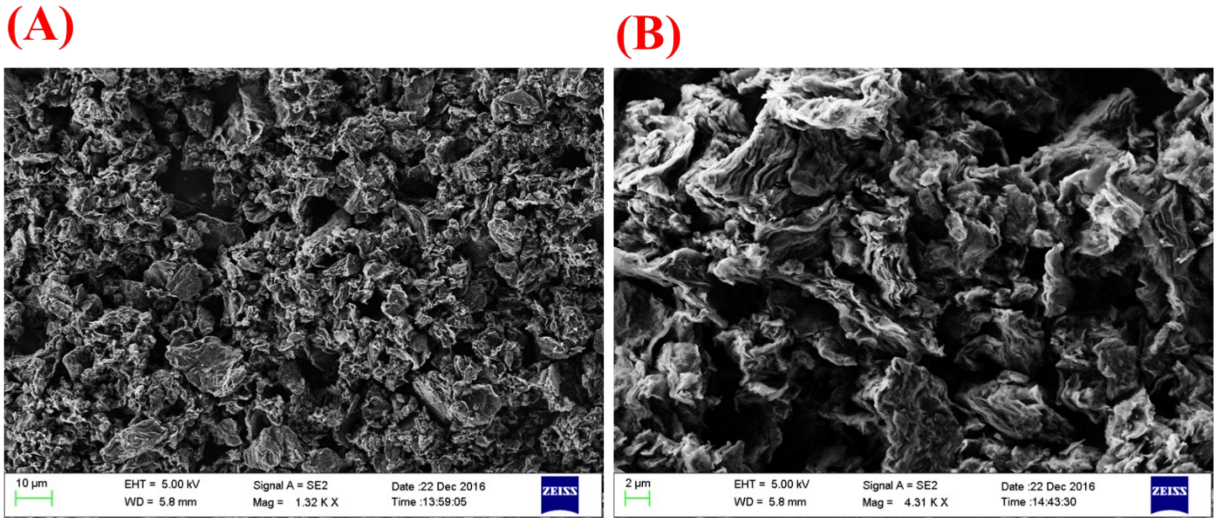

Fig. S3 FE-SEM images of the N-rGO (A-B).

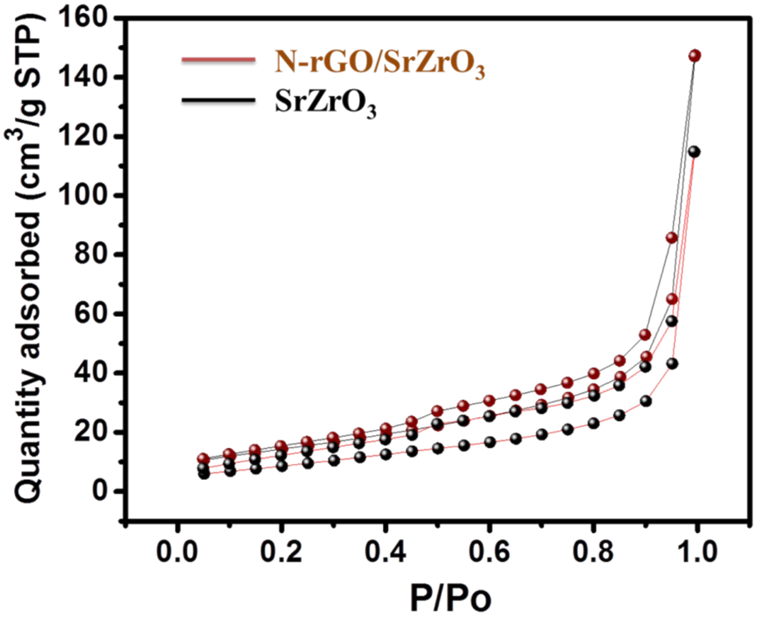

Fig. S4 N_2 adsorption/desorption of $SrZrO_3$ (black) and N-rGO/$SrZrO_3$ (brown).

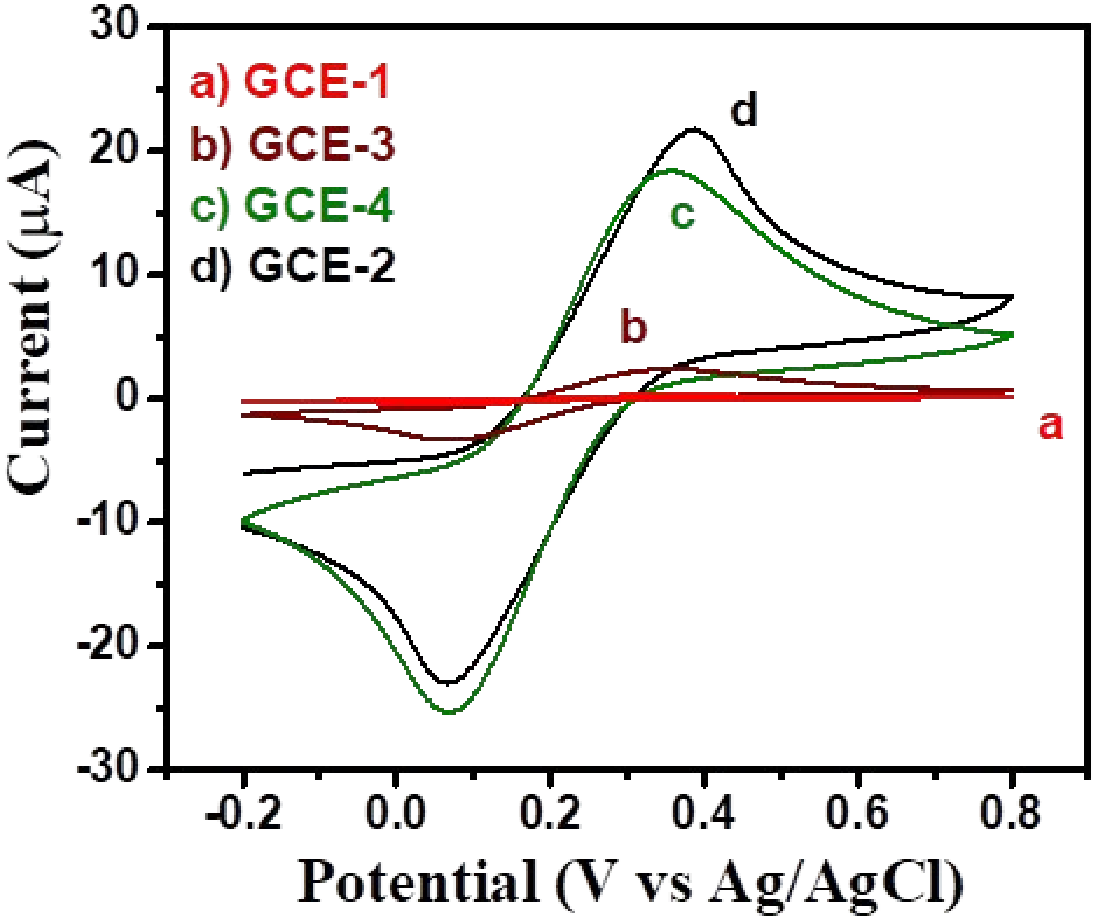

Fig. S5 CV of GCE-1 (red), **GCE-2** (black), GCE-3 (brown) and GCE-4 (green) in presence of 5mM $[Fe(CN)_6]^{3-/4-}$ at scan rate=100mV/s.

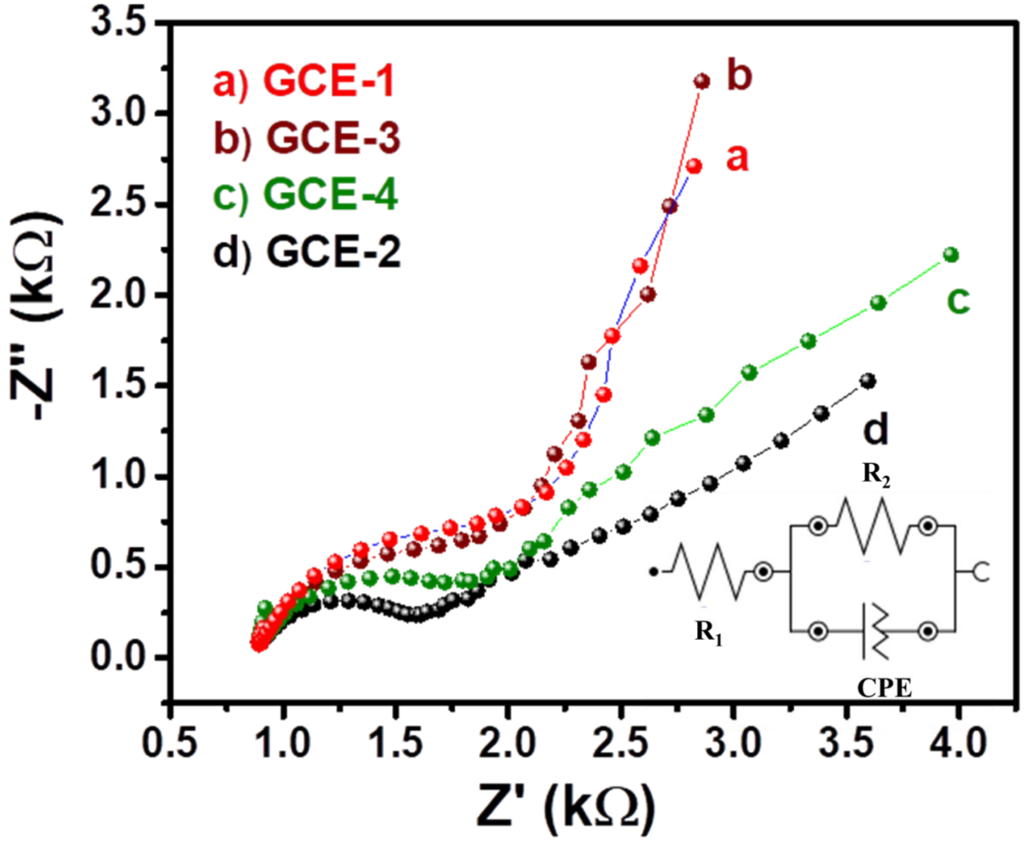

Fig. S6 Nyquist curves of GCE-1 (red), **GCE-2** (black), GCE-3 (brown) and GCE-4 (green) in presence of 5mM $[Fe(CN)_6]^{3-/4-}$ solution. Inset: Equivalent EIS circuit.

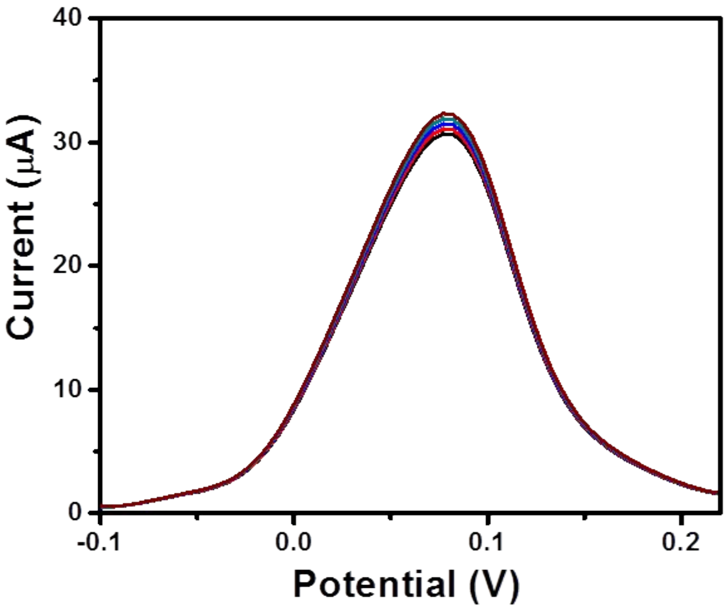

Fig. S7 Five consecutive SWV curves of **GCE-2** in 1600μM HQ in 0.1M PBS (pH=7.0).

Table S1 Crystallite parameters for $SrZrO_3$ and N-rGO/$SrZrO_3$.

S. No.	Material	2 Theta (deg)	d (ang)	Height (cps)	FWHM (deg)	Int. I (cps deg)	Size (ang)
1.	$SrZrO_3$	30.532	2.9256	1812	0.168	373	511
2.	N-rGO/$SrZrO_3$	30.504	2.9281	5765	0.197	1397	436

Table S2 EIS parameters of GCE-1, **GCE-2**, GCE-3 and GCE-4.

S.No.	Electrode	R1(Ω)	R2(Ω)
1.	GCE-1	79.9	5410
2.	**GCE-2**	62.4	1764
3.	GCE-3	77.2	4755
4.	GCE-4	68.3	1957

Where R_1= Resistance due to electrolyte

R_2= Raterials Charge transfer resistance
